# Mental Disorders Among Offspring Prenatally Exposed to Systemic Glucocorticoids

**DOI:** 10.1001/jamanetworkopen.2024.53245

**Published:** 2025-01-03

**Authors:** Kristina Laugesen, Nils Skajaa, Irene Petersen, Marianne Skovsager Andersen, Ulla Feldt-Rasmussen, Sofie Kejlberg Al-Mashhadi, Paul Stewart, Jens Otto Lunde Jørgensen, Henrik Toft Sørensen

**Affiliations:** 1 Department of Clinical Epidemiology, Department of Clinical Medicine, Aarhus University Hospital, Aarhus University, Aarhus, Denmark; 2 Department of Clinical Biochemistry, Aarhus University Hospital, Aarhus, Denmark; 3 Department of Primary Care and Population Health, University College London, London, United Kingdom; 4 Department of Endocrinology, Odense University Hospital, Odense, Denmark; 5 Department of Clinical Research, University of Southern Denmark, Odense, Denmark; 6 Department of Medical Endocrinology and Metabolism, Copenhagen University Hospital, Rigshospitalet, Copenhagen, Denmark; 7 Department of Clinical Medicine, Faculty of Health and Medical Sciences, University of Copenhagen, Copenhagen, Denmark; 8 Faculty of Medicine and Health, University of Leeds, Leeds, United Kingdom; 9 Department of Endocrinology and Internal Medicine, Aarhus University Hospital, Aarhus, Denmark

## Abstract

**Importance:**

Current evidence of the association between prenatal exposure to glucocorticoids and long-term mental disorders is scarce and has limitations.

**Objective:**

To investigate the association between prenatal exposure to systemic glucocorticoids and mental disorders in offspring at the age of 15 years, comparing exposed vs unexposed offspring born to mothers with the same underlying disease (risk of preterm delivery and autoimmune or inflammatory disorders).

**Design, Setting, and Participants:**

This nationwide population-based cohort study used data from registries in Denmark with follow-up until December 31, 2018. Participants included all Danish infants born alive from 1996 to 2016. Analyses were performed from January to December 2023.

**Exposures:**

Prenatal exposure to systemic glucocorticoids.

**Main Outcomes and Measures:**

Fifteen-year crude and adjusted risks, risk differences, and risk ratios (RR) for mental disorders using Kaplan-Meier estimator comparing exposed vs unexposed offspring born to mothers with the same underlying disease.

**Results:**

A total of 1 061 548 infants (52% male) were included in the final study cohort, including 31 518 born to mothers at risk of preterm delivery and 288 747 born to mothers with autoimmune or inflammatory disorders. Among offspring born to mothers at risk of preterm delivery, the adjusted risks for exposed vs unexposed were 6.6% vs 4.3% (RR, 1.5 [95% CI, 1.2-1.9]) for autism spectrum disorders; 1.6% vs 1.3% (RR, 1.3 [95% CI, 0.8-1.8]) for intellectual disabilities; 5.8% vs 4.3% (RR, 1.3 [95% CI, 1.0-1.7]) for attention-deficit hyperactivity disorder (ADHD); and 7.2% vs 4.6% (RR, 1.5 [95% CI, 1.1-2.0]) for mood, anxiety, and stress-related disorders. Among offspring born to mothers with autoimmune or inflammatory disorders, the adjusted risks for exposed vs unexposed were 4.8% vs 3.8% (RR, 1.3 [95% CI, 1.1-1.5]) for autism spectrum disorders; 1.1% vs 0.8% (RR 1.4, [95% CI, 0.9-2.0]) for intellectual disabilities; 5.5% vs 4.4% (RR, 1.3 [95% CI, 1.0-1.5]) for ADHD; and 6.6% vs 4.6% (RR, 1.4 [95% CI, 1.2-1.8]) for mood, anxiety, and stress-related disorders. Findings were confirmed through an active comparator and sibling design. However, confounding by disease severity could not be ruled out.

**Conclusions and Relevance:**

In this cohort study, prenatal exposure to glucocorticoids was associated with higher risk of some mental disorders. These data support continued caution in the use of glucocorticoids in pregnant people.

## Key Points

**Question:**

Is prenatal exposure to systemic glucocorticoids associated with subsequent mental disorders?

**Finding:**

In this Danish population-based cohort study of 1 061 548 infants comparing exposed vs unexposed offspring born to mothers with similar underlying disease, prenatal exposure to systemic glucocorticoids was associated with higher risk of some mental disorders.

**Meaning:**

These findings support continued caution in the use of glucocorticoids in pregnant people.

## Teaser

This cohort study investigates the association between prenatal exposure to systemic glucocorticoids and mental disorders in offspring at the age of 15 years in Denmark.

## Introduction

Systemic glucocorticoids are used in pregnant people at risk of preterm birth (to decrease neonatal morbidity and mortality) and in people with autoimmune or inflammatory disorders (to decrease inflammation and symptoms).^1^ Cortisol, an endogenous glucocorticoid, plays a critical role in normal fetal development, including development of the central nervous system (CNS).^2^ However, prenatal exposure to excess glucocorticoid levels (maternal stress or treatment) may increase the risk of mental disorders in offspring via multiple mechanisms.^2,3,4,5,6,7,8,9,10,11,12,13,14,15,16,17,18^ The evidence regarding the association with long-term mental disorders is sparse and has limitations^3,7,10,11,19,20,21^; therefore, medical and obstetric societies have called for further research on the topic.^22^

Betamethasone and dexamethasone, which pass through the placenta, are used in people at risk of preterm delivery.^1^ Randomized clinical trials^19,20,21,23^ have been constrained by either short-term follow-up (offspring ages of 2 to 10 years),^20,21,23^ small sample size (n = 82),^21^ or substantial loss to follow-up (>80%).^19^ Two observational studies have found an association of antenatal betamethasone exposure with mental disorders.^3,11^ A key limitation of those studies is their use of a general population comparator cohort, which might have conflated the effects of treatment with the effects of the underlying treated disease (confounding by indication).^3,5,6,7,10,11^ For instance, evidence suggests a link between maternal mental disorders and preterm birth, via direct pathways, genetics, or shared risk factors.^24^ These factors may also affect the risk of mental disorders in offspring. Instead, comparing exposed offspring with a cohort of unexposed offspring born to mothers with the same underlying disease may increase study validity and interpretability.^25^ Women with autoimmune or inflammatory diseases are typically treated with glucocorticoids (eg, prednisolone), which like endogenous cortisol, are inactivated by placental 11 β-hydroxysteroid dehydrogenase type 2 (11β-HSD2).^4^ Nonetheless, a small proportion of prednisolone passes the placenta, thus long-term or high-dose treatment may affect fetal development.^26^ Two observational studies have indicated that high-dose prenatal prednisolone exposure is associated with anxiety, depression, and attention-deficit hyperactivity disorders (ADHD) in offspring.^7,10^ These studies are also limited by their general population comparator cohort, which may introduce confounding by indication, shared risk factors and genetics.^27,28^ Furthermore, they assessed only a narrow spectrum of mental disorders.^7,10^

In this population-based cohort study, we examined the association between prenatal exposure to systemic glucocorticoids and mental disorders in offspring, including autism spectrum disorders, intellectual disabilities, ADHD, and mood, anxiety, and stress-related disorders. On the basis of previous animal and human studies, we hypothesized that prenatal exposure is associated with mental disorders later in life.

## Methods

This cohort study was reported as per the Strengthening the Reporting of Observational Studies in Epidemiology (STROBE) and the Reporting of Studies Conducted Using Observational Routinely-Collected Health Data (RECORD) reporting guidelines. The study was approved by the Danish Data Protection Agency. According to Danish legislation, informed consent or approval from an ethics committee is not required for registry-based studies.

### Setting and Data Sources

Denmark has a tax-supported welfare system ensuring free access to medical care. At birth or immigration, a unique identifier (the civil registration number) is assigned to each Danish resident. This number enables accurate individual-level linkage across all Danish registries as well as virtually complete follow-up.^29^ For this study, we used the Danish Medical Birth Register,^29^ the Danish National Prescription Registry,^29^ the Danish National Patient Registry,^29^ the social and demographic registers, and the Civil Registration System (Supplement 1).^29^

### Study Population

**Figure 1.**
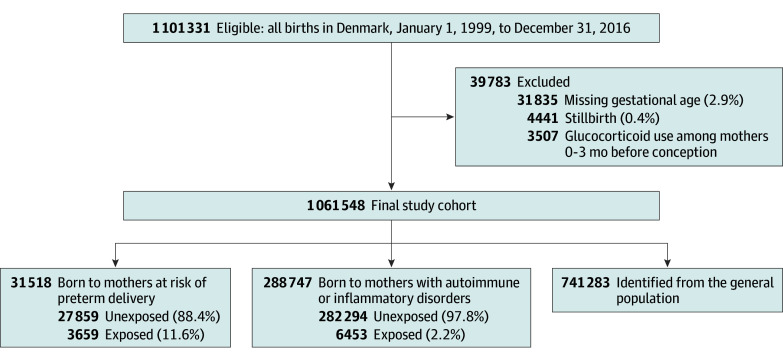
Study Flow Diagram The cohorts of offspring born to mothers at risk of preterm birth and offspring born to mothers with autoimmune or inflammatory disorders were not mutually exclusive.

We used the Medical Birth Registry to identify 1 101 331 infants born in Denmark between January 1, 1999, and December 31, 2016. We excluded stillbirths (4441 [0.4%]), infants with missing information on gestational age (31 835 [2.9]), and infants born to mothers who used systemic glucocorticoids up to 3 months before the date of conception (3507 [0.3%]), because of uncertainty about exposure status (Figure 1). Thus, the final study population consisted of 1 061 548 infants (Figure 1). We then defined 2 cohorts based on the presence of relevant indications for glucocorticoid treatment, including 31 518 infants born to mothers at risk of preterm delivery before gestational age 34 weeks, 0 days (codes recorded in the Patient Registry) (eTable 1 in Supplement 1) and 288 747 infants born to mothers with autoimmune or inflammatory disorders (ie, obstructive pulmonary disease, inflammatory bowel disease, rheumatic disease, kidney disease, skin disease, or other autoimmune or inflammatory disorders based on codes recorded in the Patient Registry; eTable 1 in Supplement 1). These cohorts were not mutually exclusive. We also defined a cohort of 741 283 infants from the general population (Figure 1).

### Prenatal Exposure

Offspring born to mothers at risk of preterm delivery were divided into those prenatally exposed and those unexposed on the basis of maternal records in the Patient Registry (eTable 2 in Supplement 1). Clinical Danish guidelines recommend 12 mg betamethasone twice, 24 hours apart. Offspring born to mothers with autoimmune or inflammatory disorders were divided in those exposed and those unexposed on the basis of a minimum of 1 prescription redeemed by the mother between the date of conception and the date of delivery (medication codes recorded in the Prescription Registry; eTable 2 in Supplement 1). Among offspring born to mothers with autoimmune or inflammatory disorders, we calculated the cumulative exposure dose during the pregnancy, expressed in prednisolone equivalents (peq) (eTable 3 in Supplement 1), and categorized this dose as less than 250 mg, 250 to 499 mg, or at least 500 mg according to the distribution. Because the cumulative exposure dose had discrete values, we did not assess the variable as a continuous variable. For offspring born to mothers with autoimmune or inflammatory disorders, we further created an additional comparison cohort of unexposed offspring born to mothers with former use of glucocorticoids (ie, people who redeemed a prescription from 3 months to 5 years before the date of conception). Finally, the general population served as an unexposed comparison cohort (Figure 1). All infants were followed-up from the date of birth until an outcome of interest, emigration, death, or the end of follow-up (December 31, 2018), whichever occurred first.

### Mental Disorders

We assessed groups of mental disorders whose onset most commonly occurs in childhood or adolescence (to correspond with our follow-up duration), including (1) intellectual disability, (2) autism spectrum disorders, (3) ADHD, and (4) mood, anxiety, or stress-related disorders. Inpatient and outpatient hospital codes recorded in the Patient Registry were used to define outcomes (eTable 1 in Supplement 1), and positive predictive values were deemed acceptable for research (65%-95%).^30,31,32^

### Covariates

Identification of potential confounders was guided by a literature review and directed acyclic graphs (eFigure 1 and eFigure 2 in Supplement 1). For offspring born to mothers with autoimmune or inflammatory disorders, we obtained information from the Medical Birth Registry regarding year of conception, parity, maternal and paternal age at conception, and maternal smoking status during pregnancy. From the Patient Registry, we obtained information on specific maternal autoimmune or inflammatory disorders; parental neurodevelopmental disorders; mood, anxiety, and stress-related disorders; schizophrenia spectrum disorders; substance use disorders; and maternal polycystic ovarian syndrome (eTable 1 in Supplement 1). Information on maternal use of comedications during pregnancy was obtained from both the Prescription and Patient Registries, including nonsteroidal anti-inflammatory drugs (NSAIDs), other immunosuppressive agents, opioids, antiepileptic medications, antidepressant agents, antipsychotic agents, and CNS stimulants (eTable 1 in Supplement 1). To measure socioeconomic status, we obtained information on the highest maternal educational level at conception (low [primary and lower secondary education], moderate [upper secondary education or professional degree], or high [university education at the bachelor’s degree level or higher]); maternal country of origin (Denmark or another country); and civil status. For offspring born to mothers at risk of preterm delivery, we additionally included pregnancy complications (gestational diabetes, preeclampsia, and maternal infections during pregnancy based on codes in the Patient and Prescription Registries; eTable 1 in Supplement 1). Pregnancy complications were not considered confounders for offspring born to mothers with autoimmune or inflammatory disorders, because complications might have occurred post exposure and have been part of the causal pathway (eFigure 2 in Supplement 1).

### Statistical Analysis

We performed the following comparisons: exposed vs unexposed offspring born to mothers at risk of preterm delivery; exposed vs unexposed offspring born to mothers with autoimmune or inflammatory disorders and; exposed vs unexposed offspring born to mothers with autoimmune or inflammatory disorders with former use of glucocorticoids. Among offspring born to mothers with autoimmune or inflammatory disorders, we also conducted dose-response analyses. We estimated crude and adjusted (weighted) 15-year absolute risks, risk differences (RD), and risk ratios (RR) by using 1 minus the Kaplan-Meier estimator. The proportion of offspring dying during follow-up was 0.9%. 95% CIs were calculated with bootstrapping (500 replications). To adjust for confounding, we used standardized morbidity ratio weighting using following steps.^33^ For each offspring in each comparison, we used the potential confounders to calculate a propensity score through a logistic regression. We then used the propensity score to reweight the comparator cohort so that the covariate distributions were similar between exposed and unexposed offspring. Compared with more conventional methods, this strategy has advantages, including estimation of adjusted absolute risks; the ability to control for numerous covariates despite smaller sample sizes and limited numbers of outcomes (eg, in dose-response and subgroup analyses); and reporting of the balance achieved between treatment and reference populations.^33^ We assessed confounder balance by using standardized differences. Analyses were performed as complete case analyses, because the proportion with missing propensity scores was less than 5%. In subgroup analyses, we stratified by sex of offspring and specific maternal autoimmune or inflammatory disorders, if the sample size permitted.

We also conducted sensitivity analyses. First, we performed a sibling-matched analysis to account for time-stable confounding, such as genetics or lifestyle. Siblings with discordant exposure were compared through conditional fixed-effect Poisson regression (separate ID for each mother). Because of the small sample size, we conducted this analysis on a composite outcome. Second, to increase the sensitivity of our recording of outcomes, we included prescriptions when appropriate (ADHD and mood, anxiety, and stress-related disorders) (eTable 1 in Supplement 1). Third, to increase confounding control in pregnant people with autoimmune or inflammatory disorders, we used an active comparator cohort, defined as pregnant people receiving other immunosuppressive agents. This analysis was restricted to offspring born to mothers with inflammatory bowel disease and rheumatic disease, who were eligible for treatment with other immunosuppressive agents, and to a composite outcome (eTable 1 in Supplement 1), owing to the limited number of outcome events available. Fourth, we performed an analysis restricted to singleton births to eliminate a potential effect of multiple pregnancies. Fifth, in the cohort of offspring born to mothers at risk of preterm delivery, we restricted an analysis to children born small for gestational age (SGA). This sensitivity analysis was performed to control for underlying fetal disease (considering SGA as a marker of intrauterine growth restriction). Six, we included SGA as a potential confounder in the cohort of offspring born to mothers at risk of preterm delivery. Seventh, to isolate glucocorticoid treatment indications, we excluded offspring born to mothers with autoimmune or inflammatory disorders from the cohort of offspring born to mothers at risk of preterm delivery and vice versa, we excluded offspring born to mothers at risk of preterm delivery from the cohort of offspring born to mothers with autoimmune or inflammatory disorders. Finally, we examined associations for exposure to specific glucocorticoids if allowed by sample size.

Statistical analyses were conducted in Stata version 17 (StataCorp). Analyses were performed from January to December 2023.

## Results

Among 1 061 548 infants (552 004 [52.0%] male) included in the final study cohort, we identified 31 518 infants (17 065 [54.4%] male) born to mothers at risk of preterm delivery (3659 [11.6%] exposed) and 288 747 infants (148 278 [51.4%] male) of mothers with autoimmune or inflammatory disorders (6453 [2.2%] exposed). The median (IQR) follow-up time was 9 (7-14) years (minimum: 2 years) for all exposure and comparison cohorts. Numbers of offspring according to maternal underlying disease and glucocorticoid treatment regimens are shown in Table 1. Parental and pregnancy characteristics are shown in Table 2; sibling characteristics are shown in eTable 4 in Supplement 1.

**Table 1.** Prenatal Exposure to Glucocorticoids Stratified by Underlying Maternal Disease

Maternal underlying disease	No. (%)						
	Risk of preterm delivery (N = 31 518)	Obstructive pulmonary disease (N = 229 707)	Inflammatory bowel disease (N = 8286)	Rheumatic disease (N = 14 961)	Kidney disease (N = 12 089)	Skin disease (N = 2886)	Other autoimmune or inflammatory disorders (N = 10 755)
Exposed	3883 (12.3)	2747 (1.2)	457 (5.5)	643 (4.3)	240 (2.0)	72 (2.5)	375 (3.5)
Cumulative dose, mg peq^a^							
<250	3778 (11.6)	1634 (0.7)	58 (0.7)	131 (0.9)	127 (1.1)	28 (1.0)	124 (1.2)
250-499	105 (0.3)	547 (0.2)	84 (1.0)	145 (1.0)	46 (0.4)	18 (0.6)	68 (0.6)
≥500	NA	566 (0.3)	315 (3.8)	367 (2.5)	67 (0.6)	26 (0.9)	183 (1.7)
Type							
Prednisolone only	160 (0.5)	1227 (0.5)	366 (4.4)	458 (3.1)	92 (0.7)	37 (1.3)	134 (1.3)
Prednisone only	42(0.1)	143 (0.06)	19 (0.2)	35 (0.2)	23 (0.2)	NA	30 (0.3)
Methylprednisolone only	NA	139 (0.06)	NA	8 (0.05)	7 (0.05)	NA	11 (0.1)
Betamethasone only	3659 (12)	1146 (0.5)	49 (0.6)	105 (0.7)	119 (0.9)	24 (0.8)	115 (1.1)
Dexamethasone only	NA	NA	NA	NA	NA	NA	NA
Hydrocortisone only	NA	30 (0.01)	NA	NA	NA	NA	70 (0.7)
Triamcinolone only	NA	NA	NA	NA	NA	NA	NA
Multiple types	22 (0.07)	46 (0.02)	14 (0.2)	33 (0.2)	8 (0.06)	NA	15 (0.14)

Abbreviations: NA, not available because of Danish legislation regarding individual-level data; peq, prednisone equivalents.

^a^ The cumulative systemic glucocorticoid dose in peq was calculated by multiplication of the number of pills/injections, dose per pill/injection, and prednisolone conversion factor for cumulative prescriptions during pregnancy.

**Table 2.** Parental and Pregnancy Characteristics of Exposed and Unexposed Offspring Born to Mothers With the Same Underlying Disease^a^

Characteristics	No. (%)					
	Risk of preterm delivery			Autoimmune or inflammatory disorders		
	Exposed	Unexposed		Exposed	Unexposed	
		Unweighted	Weighted		Unweighted	Weighted
All births	3659 (100)	27 859 (100)	3349 (100)	6453 (100)	282 294 (100)	6010 (100)
Offspring sex						
Male	1901 (52.0)	15 164 (54.4)	1828 (54.5)	3323 (51.5)	144 955 (51.3)	3071 (51.1)
Female	1758 (48.0)	12 695 (45.6)	1521 (45.4)	3130 (48.5)	137 339 (48.7)	2939 (48.9)
Year of conception						
1999-2002	680 (18.6)	7985 (28.7)	534 (16.0)	951 (14.7)	61 511 (21.7)	980 (16.3)
2003-2006	732 (20.0)	6675 (24.0)	640 (19.1)	1353 (21.0)	62 308 (22.1)	1195 (19.9)
2007-2010	900 (25.0)	6404 (23.0)	863 (25.8)	1720 (26.7)	68 507 (24.3)	1517 (25.2)
2011-2015	1347 (36.8)	6795 (24.4)	1312 (39.2)	2429 (37.6)	89 968 (31.8)	2319 (38.6)
Maternal characteristics						
Age at birth, median (IQR), y	31 (28-35)	30 (26-33)	31 (28-35)	32 (28-36)	30 (27-34)	32 (28-36)
Parity						
0	2338 (63.9)	16 021 (57.5)	2146 (64.1)	3611 (56.0)	118 855 (42.1)	3381 (56.3)
≥1	1305 (35.7)	11 393 (40.9)	1203 (35.9)	2771 (42.9)	160 330 (56.8)	2630 (43.8)
Missing	16 (0.4)	445 (1.6)	0	71 (1.1)	3109 (1.1)	0
Country of origin						
Denmark	2749 (75.1)	23,643 (84.9)	2545 (76.0)	5462 (84.6)	252 608 (89.4)	5164 (85.9)
Missing	58 (0.9)	178 (0.7)	0	45 (0.7)	1327 (0.5)	0
Highest educational level^b^						
Low	549 (15.0)	6208 (22.3)	501 (15.0)	1057 (16.4)	57 577 (20.4)	961 (16.0)
Moderate	1290 (35.3)	12 055 (43.3)	1270 (37.9)	2710 (42.0)	121 595 (43.1)	2625 (43.7)
High	1710 (46.7)	8900 (31.9)	1578 (47.1)	2608 (40.4)	98 005 (34.7)	2425 (40.3)
Missing	110 (3.0)	696 (2.5)	(0.0)	78 (1.2)	5117 (1.8)	0 (0)
Marital status						
Married/civil partnership	2073 (57)	15 435 (55)	1944 (58)	3834 (59)	160 036 (57)	3623 (60)
Missing	69 (1.9)	246 (0.9)	0	68 (1.0)	1861 (0.7)	0
BMI, median (IQR)						
<18.5	216 (6.5)	1226 (6.2)	147 (5.2)	235 (4.3)	8828 (4.0)	185 (3.7)
18.5-24	2178 (65.1)	11 644 (58.5)	1800 (64.0)	3216 (58.4)	123 266 (55.8)	2905 (57.7)
25-29	499 (14.9)	3722 (18.7)	422 (15.0)	1123 (20.4)	47 896 (21.7)	1084 (21.6)
≥30	314 (9.4)	2263 (11.4)	315 (11.2)	720 (13.1)	33 002 (14.9)	705 (14.0)
Missing (2004 onward)	136 (4.1)	1019 (5.1)	118 (4.4)	208 (3.8)	7791 (3.5)	152 (3.0)
Not recorded before 2004	316 (100)	7938 (100)	534 (100)	951 (100)	61 511 (100)	980 (100)
Smoking during pregnancy	495 (13.5)	5441 (19.5)	529 (15.8)	886 (13.7)	53 322 (18.8)	926 (15.4)
Obstructive pulmonary disease	743 (20.3)	7018 (25.2)	686 (20.5)	2747 (42.6)	226 960 (80.3)	2577 (42.9)
Inflammatory bowel disease	25 (0.7)	285 (1.0)	22 (0.7)	457(7.1)	7829 (2.8)	473 (7.9)
Rheumatic disease	57 (1.6)	498 (1.8)	48 (1.5)	643 (10)	14 318 (5.1)	611 (10)
Kidney disease	84 (2.3)	503 (1.8)	78 (2.3)	240 (3.7)	11 849 (4.2)	226 (3.8)
Skin disease	11 (0.3)	88 (0.3)	11 (0.3)	72 (1.1)	2814 (1.0)	72 (1.2)
Neurodevelopmental disorders	26 (0.7)	227 (0.8)	23 (0.7)	53 (0.8)	2861 (1.0)	49 (0.8)
Mood, anxiety, or stress-related disorders	648 (17.7)	5032 (18.1)	602 (18.0)	1333 (20.6)	55 560 (19.7)	1262 (21.0)
Schizophrenia spectrum disorders	104 (2.8)	1078 (3.9)	96 (2.9)	279 (4.3)	10 911 (3.9)	258 (4.3)
Substance use disorders	34 (0.9)	238 (0.9)	31 (0.9)	48 (0.7)	2227 (0.8)	41 (0.7)
PCOS	75 (2.1)	543 (2.0)	72 (2.1)	156 (2.4)	3716 (1.3)	149 (2.5)
Co-medication use during pregnancy						
Other immunosuppressive agents	8 (0.2)	70 (0.3)	8 (0.2)	309 (4.8)	1499 (0.5)	325 (5.4)
NSAIDs	43 (1.2)	580 (2.1)	40 (1.2)	281 (4.4)	5689 (2.0)	263 (4.4)
Antiepileptic medications	13 (0.4)	119 (0.4)	11 (0.3)	40 (0.6)	1182 (0.4)	40 (0.7)
Opioids	28 (0.7)	283 (1.0)	28 (0.8)	156 (2.4)	2947 (1.0)	150 (2.5)
Antidepressants	76 (2.1)	689 (2.5)	71 (2.1)	191 (3.0)	5877 (2.1)	184 (3.1)
Antipsychotics	7 (0.2)	94 (0.3)	6 (0.2)	25 (0.4)	704 (0.3)	24 (0.4)
Stimulants	NA	25 (0.1)	NA	10 (0.2)	327 (0.1)	9 (0.2)
Paternal characteristics						
Age at birth, median (IQR), y	33 (30-37)	32 (28-36)	33 (30-37)	34 (30-38)	32 (29-36)	34 (30-38)
Neurodevelopmental disorders	25 (0.7)	313 (1.1)	23 (0.7)	62 (0.9)	3272 (1.2)	60 (1.0)
Mood, anxiety, or stress-related disorders	290 (7.9)	2495 (9.0)	274 (8.2)	685 (11)	27 904 (9.9)	665 (11)
Schizophrenia spectrum disorders/psychoses	82 (2.2)	797 (2.9)	73 (2.2)	193 (3.0)	8289 (2.9)	188 (3.1)
Substance use disorders	28 (0.8)	322 (1.2)	26 (0.8)	65 (1.0)	3271 (1.2)	64 (1.0)
Pregnancy complications						
Multiple pregnancy	1297 (35.4)	7343 (26.4)	1189 (35.5)	1012 (15.7)^c^	11 055 (3.9)	293 (5.0)^c^
Gestational diabetes	136 (3.7)	730 (2.6)	130 (3.9)	268 (4.2)^c^	6581 (2.3)	174 (2.9)^c^
Preeclampsia	409 (11.2)	1098 (3.9)	373 (11.1)	431 (6.7)^c^	9030 (3.2)	230 (3.8)^c^
Infections	802 (21.9)	6575 (23.6)	732 (21.9)	2111 (32.7)^c^	78 717 (27.9)	1646 (27.4)^c^

Abbreviations: BMI, body mass index (calculated as weight in kilograms divided by height in meters squared); NSAIDs, nonsteroidal anti-inflammatory drugs; PCOS, polycystic ovarian syndrome; SGA, small for gestational age.

^a^ Standardized differences are all less than or equal to 0.1 and are not shown because of Danish legislation regarding individual-level data.

^b^ Highest educational level at birth: low (primary and lower secondary education), moderate (upper secondary education or professional degree), and high (university education at bachelor’s degree level or higher.

^c^ Variables considered potential mediators (eFigure 2 in Supplement 1), thus not included in the propensity score weighting. The variables are not expected to be balanced after weighting.

### Characteristics of Offspring Born to Mothers at Risk of Preterm Delivery

Compared with unexposed offspring born to mothers at risk of preterm delivery, exposed offspring had mothers with a higher prevalence of maternal pregnancy complications (Table 2). Mothers of exposed offspring were slightly older, had higher educational achievement, were more likely to originate from a country other than Denmark, and had a lower prevalence of smoking (14% vs 20%) (Table 2). After weighting, the distributions of all confounders were similar between exposed and unexposed (Table 2), and the standardized differences were all less than or equal to 0.1 (not shown because of Danish legislation regarding individual-level data).

### Characteristics of Offspring Born to Mothers With Autoimmune or Inflammatory Disorders

Compared with unexposed offspring born to mothers with autoimmune or inflammatory disorders, exposed offspring had mothers with a higher prevalence of inflammatory bowel disease (7.1% vs 2.8%) and rheumatic disease (19% vs 5.1%) but a lower prevalence of obstructive pulmonary disease (43% vs 80%). Mothers of exposed offspring were slightly older, had higher educational achievement, and had a lower prevalence of smoking (14% vs 19%). After weighting, the distributions of all confounders were similar between exposed and unexposed (Table 2), and the standardized differences were all less than or equal to 0.1.

#### Association in Offspring Born to Mothers at Risk of Preterm Delivery

**Figure 2.**
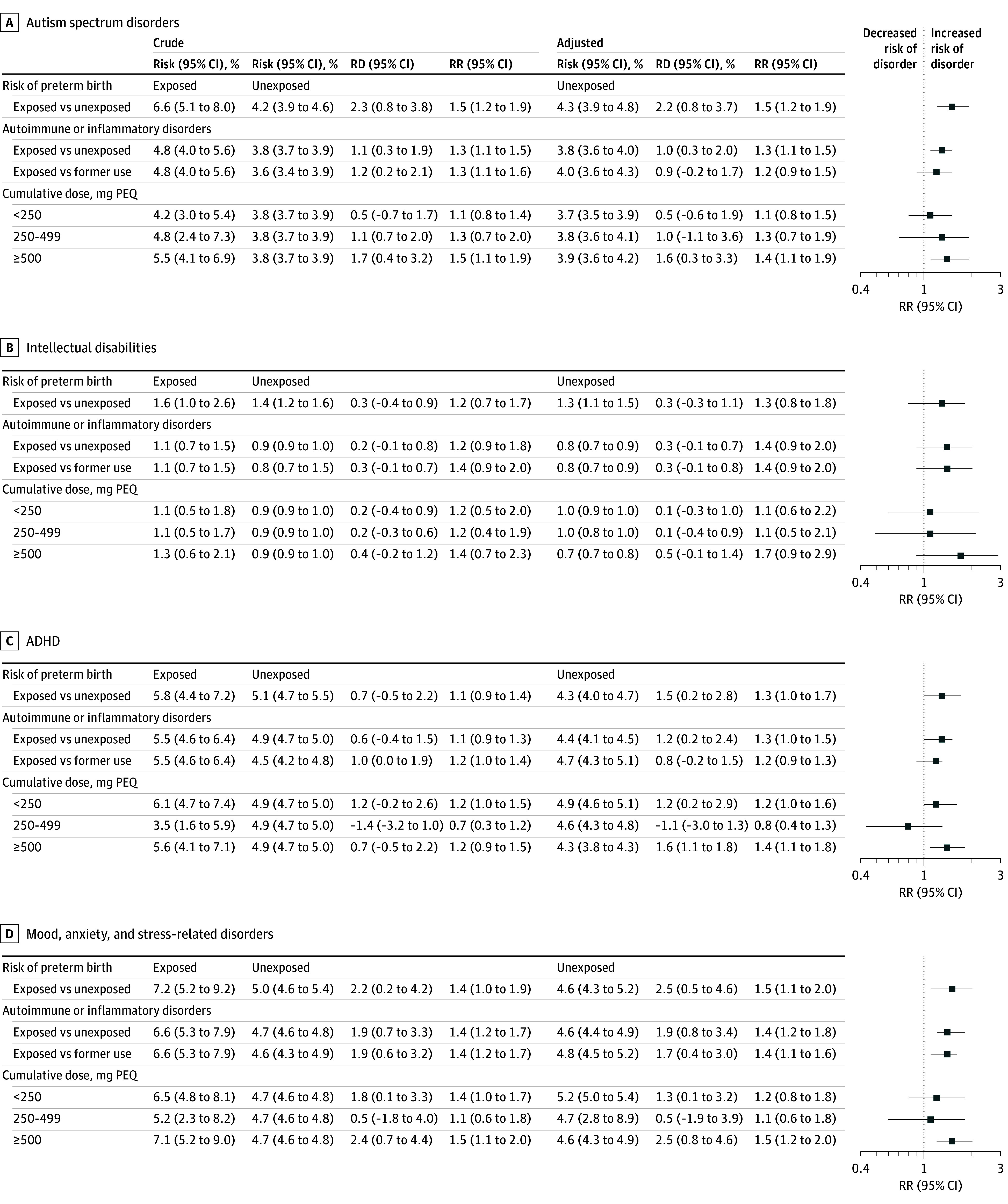
Fifteen-Year Risks, Risk Differences, and Relative Risks for Mental Disorders Adjusted for year of conception; parity; maternal and paternal age at conception; maternal smoking during pregnancy; specific maternal autoimmune or inflammatory disorders; maternal and paternal neurodevelopmental disorders; maternal and paternal mood, anxiety, and stress-related disorders; maternal and paternal schizophrenia spectrum disorders; maternal and paternal substance use disorders; maternal polycystic ovarian syndrome; maternal use of comedications during pregnancy, including nonsteroidal anti-inflammatory drugs, other immunosuppressive agents, opioids, antiepileptic medications, antidepressants, antipsychotics, and stimulants; highest maternal educational level at conception; maternal country of origin; and civil status. For offspring born to mothers at risk of preterm delivery, we additionally adjusted for singleton/multiple pregnancy, gestational diabetes, preeclampsia, and maternal infections during pregnancy. Underlying number at risk and number of outcomes are provided in eTable 5 in Supplement 1. Kaplan-Meier curves are not presented due to Danish legislation regarding individual-level data. ADHD indicates attention deficit hyperactivity disorder; PEQ, prednisolone equivalents; RD, risk difference; RR, relative risk.

Point estimates for all 4 groups of mental disorders were higher for prenatal exposure to glucocorticoids (Figure 2; eTable 5 in Supplement 1), although 95% CIs for intellectual disabilities crossed 1.0. When comparing exposed vs unexposed offspring born to mothers at risk of preterm delivery the adjusted 15-year risks were 6.6% vs 4.3% (aRR, 1.5 [95% CI, 1.2-1.9]) for autism spectrum disorders; 1.6% vs 1.3% (aRR, 1.3 [95% CI, 0.8-1.8]) for intellectual disabilities; 5.8% vs 4.3% (aRR, 1.3 [95% CI, 1.0-1.7]) for ADHD; and 7.2% vs 4.6% (aRR, 1.5 [95% CI, 1.1-2.0]) for mood, anxiety, and stress-related disorders (Figure 2).

#### Association in Offspring Born to Mothers With Autoimmune or Inflammatory Disorders

Point estimates for all 4 groups of mental disorders were higher for prenatal exposure to glucocorticoids (Figure 2; eTable 5 in the Supplement), although 95% CIs for intellectual disabilities crossed 1.0. When comparing exposed vs unexposed offspring born to mothers with autoimmune or inflammatory disorders, the 15-year adjusted risks were 4.8% vs 3.8% (aRR, 1.3 [1.1-1.5]) for autism spectrum disorders; 1.1% vs 0.8% (aRR, 1.4 [95% CI, 0.9-2.0]) for intellectual disabilities; 5.5% vs 4.4% (aRR of 1.3 [95% CI, 1.0-1.5]) for ADHD; and 6.6% vs 4.6% (aRR of 1.4 [95% CI, 1.2-1.8]) for mood, anxiety, and stress-related disorders (Figure 2). Associations persisted when exposed offspring were compared with unexposed offspring born to mothers with former glucocorticoid use.

#### Sensitivity and Subgroup Analyses

Estimates were robust in the sensitivity analyses (eTable 6, eTable 7, and eTable 8 in Supplement 1). For risk of preterm birth, the sibling-matched analysis yielded an aRR of 1.4 (95% CI, 0.5-3.9) for the composite outcome. This analysis was based on 139 exposed and 129 unexposed offspring (129 sibling pairs born to 129 mothers). For offspring born to mothers with autoimmune or inflammatory disorders, the sibling-matched analysis yielded an aRR of 1.3 (95% CI, 1.0-1.6) based on 2437 exposed and 2805 unexposed offspring (2437 sibling pairs born to 2437 mothers). Use of an active comparator cohort for offspring born to mothers with inflammatory bowel disease or rheumatic disease (ie, other immunosuppressive agents) yielded an aRR of 1.7 (95% CI, 1.1-3.5) for the composite outcome (eTable 6 in Supplement 1). Restricting analyses to singleton births, offspring born SGA, risk of preterm birth only, and to autoimmune or inflammatory disorders only, respectively, yielded similar results as the main analyses (eTable 6 and eTable7 in Supplement 1). Finally, increasing completeness of detection of ADHD and mood, anxiety, and stress-related disorders by adding medication use to the outcome definition did not alter results (eTable 6 in Supplement 1).

Stratification by sex of offspring only slightly affected the associations and did not show a clear pattern (eTable 9 in Supplement 1). The associations increased when the general population was used as a comparator (eTable 10 in Supplement 1).

## Discussion

Our cohort study found an association between prenatal exposure to systemic glucocorticoids and mental disorders later in life. This finding adds to the limited prior research in multiple ways. Importantly, compared with former observational studies, we aimed to decrease confounding and other types of bias stemming from maternal health and health-seeking behavior associated with the glucocorticoid treatment indication. We addressed this issue by comparing offspring with vs without glucocorticoid exposure born to mothers with the same underlying disease. This approach led to a slight attenuation of the associations observed with the use of a general population comparator. In people at risk of preterm delivery, we confirmed the findings of Räikkönen et al.^3^ In contrast to Räikkönen et al, we refrained from adjusting for and stratifing by covariates in the potential causal pathway (Apgar score, birthweight, care in a neonatal intensive unit, and preterm birth). Adjusting for or stratifying by mediators is not recommended, because such an approach can lead to collider bias.^34^ In pregnant people with autoimmune or inflammatory disorders, we confirmed our previous findings of an association between prenatal exposure to high-dose prednisolone and anxiety, depression, and ADHD.^7,10^ The randomized clinical trial by Dalziel et al^19^ reported 6 individuals with mental disorders among 87 betamethasone exposed and 6 among 105 placebo exposed (7% vs 6%; relative risk, 1.2 [95% CI, 0.40-3.6]) during 31 years of follow up. Compared with our study, this study was limited by more than 80% of participants being lost to follow-up and insufficient sample size for outcome detection.

Furthermore, our findings support those of previous animal and human studies. Mechanistically, the theory of fetal programming may be important.^2,8,9,12^ First, exposure may alter CNS structure and function.^13,14,15,35,36,37^ For example, animal studies have shown that prenatal administration delays both astrocyte and capillary tight junction maturation, and myelination of the corpus callosum in fetal sheep.^35,36^ Furthermore, administration has been reported to affect the neuronal cytoskeleton and presynaptic terminals in baboons.^37^ Human studies have reported larger amygdala volume, altered neuronal connectivity, and altered CNS receptor density after prenatal exposure.^13,14,15^ Second, prenatal exposure may modify the regulation of the hypothalamic-pituitary-adrenal axis (stress axis) toward greater bursts of cortisol.^8,9,12^ Finally, administered glucocorticoids can suppress maternal production of cortisol.^2^ Cortisol and synthetic glucocorticoids display different receptor affinities and may disrupt natural development.

Our data support continued caution in the use of glucocorticoids in pregnant people. However, our findings should be balanced against the risks of leaving people untreated and viewed in light of low to moderate absolute risk differences. Short-term benefits of systemic glucocorticoid treatment are multiple. However, future research could focus on reducing use in pregnant people. Among people at risk of preterm delivery, some give birth at term and thus are exposed to unnecessary treatment. A recent meta-analysis has reported this proportion to be 40%.^38^ In our population, the proportion was smaller (17%). Future research could focus on improving preterm birth risk stratification to avoid unnecessary exposure to people who ultimately give birth at term. In people with autoimmune or inflammatory disorders, alternative drugs might be safer and could potentially decrease the use of glucocorticoids, but evidence is lacking and more research is needed. Research could also benefit from follow-up into adulthood.

### Limitations

Our study has limitations. First, we restricted our study population to live births. Second, we cannot rule out the possibility of confounding. Despite using multiple approaches to reduce confounding from the well-known association between mental disorders and both autoimmune disorders and preterm birth,^24,27,28^ this bias cannot be ruled out. For instance, the sibling design and our approach of comparing offspring with vs without exposure born to mothers with the same underlying disease cannot control for confounding from disease severity. However, comparisons with an active comparator did not attenuate the associations. Third, information on paternal civil registration numbers was missing for some offspring, thus leading to incomplete registration of paternal educational level and health. Fourth, we cannot account for factors occurring during the upbringing of offspring, such as chronic health disorders, trauma, or other circumstances affecting mental health. Fifth, we cannot dismiss nondifferential misclassification of the exposure; for instance, medication adherence is not measured. Misclassification might have led to bias toward the null in the overall comparison. The hospital-based outcome definitions were previously validated with positive predictive values acceptable for research.^30,31,32^ Finally, median (IQR) follow-up time was 9 (7-14) years with a minimum of 2 years. Further follow up is needed to fully examine the associations.

## Conclusions

In this cohort study, prenatal exposure to glucocorticoids was associated with mental disorders. Our findings should be balanced against the risks of leaving pregnant people untreated and viewed in light of low to moderate absolute risk differences. Furthermore, confounding by disease severity cannot be ruled out. Our data support continued caution in the use of glucocorticoids in pregnant people.

## Notes

Supplement 1. **eFigure 1.** Directed Acyclic Graph for Offspring Born to Women at Risk of Preterm Delivery **eFigure 2.** Directed Acyclic Graph for Offspring Born to Women With Autoimmune or Inflammatory Disorders **eTable 1.** Codes Used to Define Cohorts, Covariates, and Outcomes **eTable 2.** Anatomical Therapeutic Chemical (ATC) Classification and Procedure Codes for Use of Relevant Drugs **eTable 3.** Equivalency Table Presenting Systemic Glucocorticoid Doses and Corresponding Prednisolone Conversion Factors **eTable 4.** Parental and Pregnancy Characteristics of Exposed and Unexposed Siblings **eTable 5.** Numbers at Risk and Numbers of Outcomes According to Exposure in Unweighted and Weighted Cohorts **eTable 6.** Results of Sensitivity Analyses **eTable 7.** Results of Sensitivity Analyses **eTable 8.** Results of Sensitivity Analyses **eTable 9.** Results of Subgroup Analyses **eTable 10.** Adjusted 15-Year Relative Risks and 95% Confidence Intervals for Exposed Offspring vs All Unexposed Offspring from the General Population

Supplement 2.
Data Sharing Statement
