# Errors in Article Information

**DOI:** 10.1001/jamanetworkopen.2025.1744

**Published:** 2025-02-21

In the Original Investigation titled “Mental Disorders Among Offspring Prenatally Exposed to Systemic Glucocorticoids,”^1^ published January 3, 2025, the names of Profs Andersen, Jørgensen, and Sørensen appeared incorrectly in the Article Information section. This article has been corrected.^1^
