# Supplementary material for: Mental Disorders Among Offspring Prenatally Exposed to Systemic Glucocorticoids
Source: JAMA Netw Open. 2025 Jan 3;8(1):e2453245. doi: 10.1001/jamanetworkopen.2024.53245 (PMC11699534; doi:10.1001/jamanetworkopen.2024.53245)
Supplement: Supplement 1. — eFigure 1. Directed Acyclic Graph for Offspring Born to Women at Risk of Preterm Delivery eFigure 2. Directed Acyclic Graph for Offspring Born to Women With Autoimmune or Inflammatory Disorders eTable 1. Codes Used to Define Cohorts, Covariates, and Outcomes eTable 2. Anatomical Therapeutic Chemical (ATC) Classification and Procedure Codes for Use of Relevant Drugs eTable 3. Equivalency Table Presenting Systemic Glucocorticoid Doses and Corresponding Prednisolone Conversion Factors eTable 4. Parental and Pregnancy Characteristics of Exposed and Unexposed Siblings eTable 5. Numbers at Risk and Numbers of Outcomes According to Exposure in Unweighted and Weighted Cohorts eTable 6. Results of Sensitivity Analyses eTable 7. Results of Sensitivity Analyses eTable 8. Results of Sensitivity Analyses eTable 9. Results of Subgroup Analyses eTable 10. Adjusted 15-Year Relative Risks and 95% Confidence Intervals for Exposed Offspring vs All Unexposed Offspring from the General Population [file jamanetwopen-e2453245-s001.pdf]

Supplemental Online Content

Laugesen K, Skajaa N, Petersen I, et al. Mental disorders among offspring prenatally exposed to systemic glucocorticoids. *JAMA Netw Open.* 2025;8(1):e2453245. doi:10.1001/jamanetworkopen.2024.53245

eFigure 1. Directed Acyclic Graph for Offspring Born to Women at Risk of Preterm Delivery

eFigure 2. Directed Acyclic Graph for Offspring Born to Women With Autoimmune or Inflammatory Disorders

eTable 1. Codes Used to Define Cohorts, Covariates, and Outcomes

eTable 2. Anatomical Therapeutic Chemical (ATC) Classification and Procedure Codes for Use of Relevant Drugs

eTable 3. Equivalency Table Presenting Systemic Glucocorticoid Doses and Corresponding Prednisolone Conversion Factors

eTable 4. Parental and Pregnancy Characteristics of Exposed and Unexposed Siblings

eTable 5. Numbers at Risk and Numbers of Outcomes According to Exposure in Unweighted and Weighted Cohorts

eTable 6. Results of Sensitivity Analyses

eTable 7. Results of Sensitivity Analyses

eTable 8. Results of sensitivity analyses.

eTable 9. Results of Subgroup Analyses

eTable 10. Adjusted 15-Year Relative Risks and 95% Confidence Intervals for Exposed Offspring vs. All Unexposed Offspring from the General Population

This supplemental material has been provided by the authors to give readers additional information about their work.

© 2025 Laugesen K et al. *JAMA Network Open.*

Description of data sources

- The Danish Medical Birth Register, which contains information on all births in Denmark since 1973. Information includes the civil registration numbers of the infant, mother, and father; information associated with the pregnancy and delivery; and infant and maternal characteristics.
- The Danish National Prescription Registry, which contains information on all prescriptions redeemed at community pharmacies since 1995, coded according to the Anatomical Therapeutic Chemical Classification System (ATC).
- The Danish National Patient Registry, which contains information on all patients discharged from all Danish non-psychiatric hospitals since 1977. Since 1995, this registry has also included data on contacts with hospital psychiatric departments, emergency departments, and hospital outpatient clinics. Diagnoses are coded according to the International Classification of Diseases (Eighth Revision [ICD-8] between 1977 and 1993 and Tenth Revision [ICD-10] thereafter). This registry also contains information on the use of selected types of medications dispensed in-hospital, including glucocorticoid treatment for imminent preterm delivery (but not for other treatment indications) and treatment with other immunosuppressive agents.
- The social and demographic registers contain longitudinal data since 1980 on family, income, education, and employment.
- The Civil Registration System, which contains information on all Danish residents since 1968, including date of birth, sex, immigration/emigration, and vital status.

© 2025 Laugesen K et al. *JAMA Network Open.*

eFigure 1. Directed acyclic graph for offspring born to women at risk of preterm delivery.

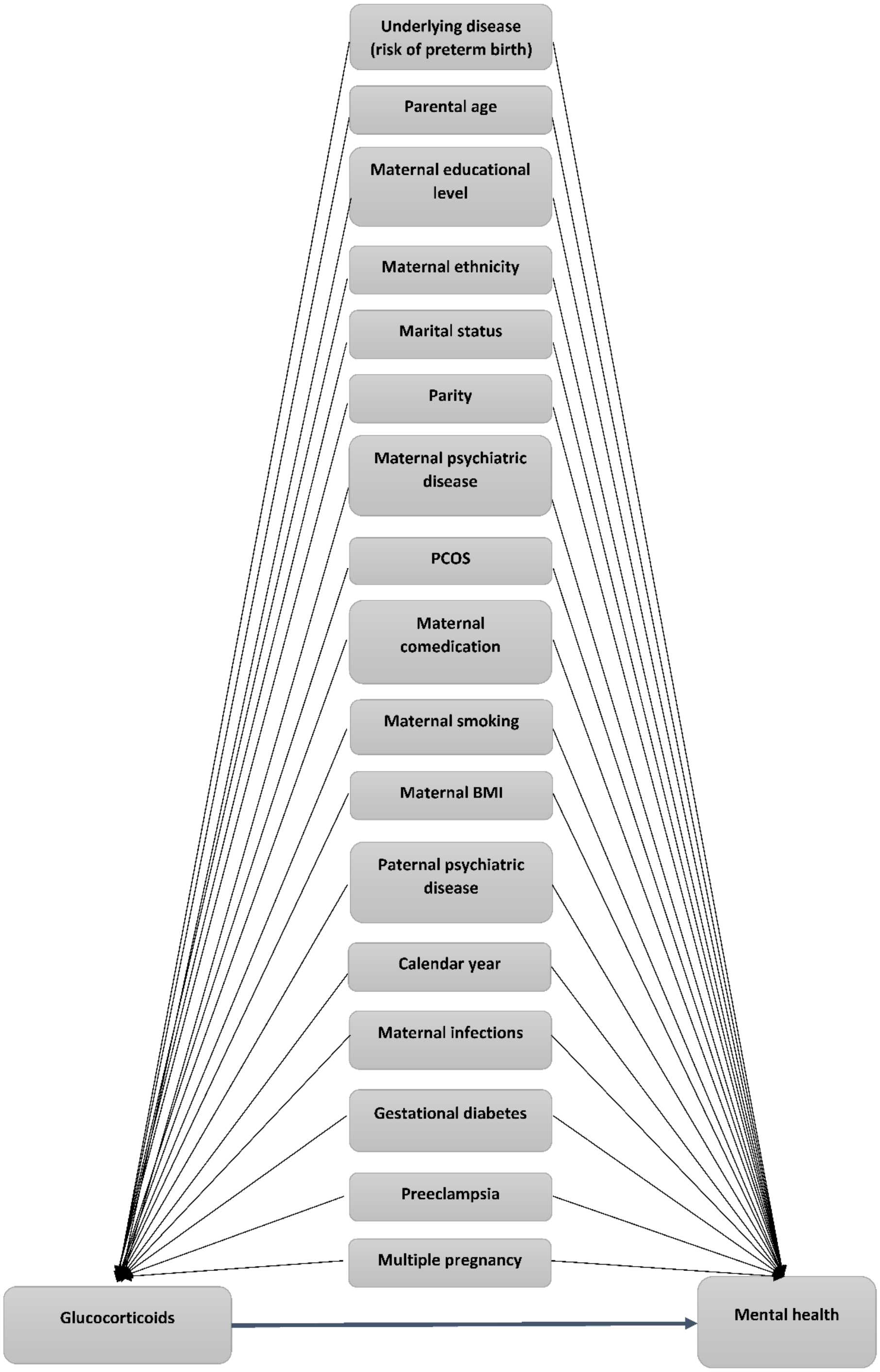

Note: Maternal comedication use during pregnancy includes other immunosuppressive agents, nonsteroidal anti-inflammatory drugs, opioids, antidepressants, antipsychotics, stimulants, and antiepileptic drugs.

© 2025 Laugesen K et al. *JAMA Network Open.*

eFigure 2. Directed acyclic graph for offspring born to women with autoimmune or inflammatory disorders.

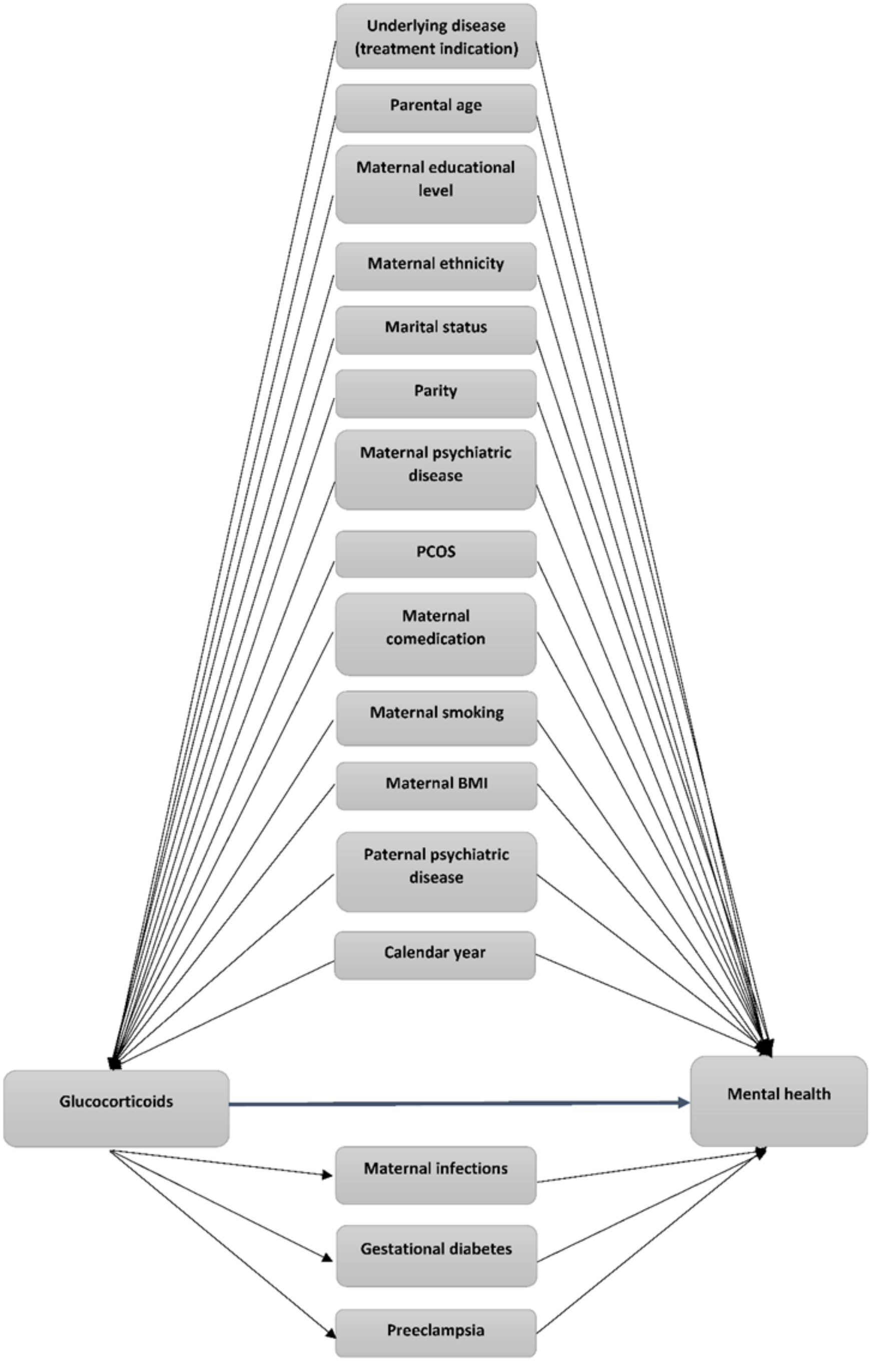

Note: Maternal underlying diseases (treatment indications) include obstructive pulmonary diseases, inflammatory bowel diseases, rheumatic diseases, renal diseases, skin diseases, and others. Maternal comedication use during pregnancy includes other immunosuppressive agents, nonsteroidal anti-inflammatory drugs, opioids, antidepressants, antipsychotics, stimulants, and antiepileptic drugs.

© 2025 Laugesen K et al. *JAMA Network Open.*

eTable 1. Codes used to define cohorts, covariates, and outcomes.

	ICD-8	ICD-10 or procedure codes	ATC/procedure
**Mental health outcomes**			
Autism spectrum disorders	NA	F84.x, excluding F842–F844	
Intellectual disability	NA	F7	
ADHD	NA	F90	N06BA
Mood, stress-related, or anxiety disorders	NA	F30–48, F91, F93	N06A, N05AN
Composite outcome		F7, F30–48, F84.x, excluding F842–F844, F90, F91, F93	N06A, N05AN, N06BA
**Cohort establishment and covariates**			
Risk of preterm delivery	NA	O470, O472, O422, O424, O60	
Obstructive pulmonary disease	491, 492, 493	J40–J46	R03
Inflammatory bowel disease	56301, 56302, 56309, 56319	K50, K51	
Rheumatic disease	446, 712, 716, 696, 734, 69549	M30–M36 M05–M14, M45, L93, L94, N085, N164, D690, I776, L95	
Renal disease	403, 404, 580–583, 59320	N00–08, N10–N12, N14–N16	
Skin disease	694, 697, 69610, 69619	L10–L14, L40, L43	
Other autoimmune disease (hematological, endocrine, neurological, or gastrointestinal)	28390, 28710, 24200, 24201, 24208, 24209, 24401, 24503, 25510, 340, 73309, 71249	D590, D591, D693, E050, E063, E271–3, G35, G70, K732	
Imminent preterm delivery		O472, O420, O422, O424, O60	BBHF3
Neurodevelopmental disorders	NA	F7, F80–90	N06BA
Mood, anxiety, OCD, or stress-related disorders or use of antidepressants	NA	F30–F48, F91, F93	N06A, N05AN

© 2025 Laugesen K et al. *JAMA Network Open.*

Schizophrenia spectrum disorders or use of antipsychotics	NA	F20–F29	N05A (except N05AN)
Substance use disorders	NA	F10–F19	
PCOS	25690	E282	
**Co-medication use during pregnancy**			
Antiepileptic medications			N03
Opioids			N02A
NSAIDs			M01
Other immunosuppressive		BOHJ	L04<br>P01BA02<br>A07EC0
Antidepressants or mood stabilizers			N06A, N05AN
Antipsychotics			N05A (except N05AN)
Central nervous system stimulants			N06BA
**Pregnancy complications**			
Gestational diabetes		O244	
Preeclampsia		O14–O15	
Infections during pregnancy		O23, A00–A99, B00–B99	J01–J05

Abbreviations: ATC, anatomic, therapeutic, chemical. ICD, *International Classification of Diseases*. NSAID, non-steroidal anti-inflammatory drugs.

© 2025 Laugesen K et al. *JAMA Network Open.*

eTable 2. Anatomical Therapeutic Chemical (ATC) Classification and procedure codes for use of relevant drugs.

Medication type	ATC codes	Procedure codes
Systemic glucocorticoids (oral or injectable)		
Betamethasone	H02AB01	BBHF3
Dexamethasone	H02AB02	
Methylprednisolone	H02AB04	
Prednisolone	H02AB06	
Prednisone	H02AB07	
Triamcinolone	H02AB08	
Hydrocortisone	H02AB09	

© 2025 Laugesen K et al. *JAMA Network Open.*

eTable 3. Equivalency table presenting systemic glucocorticoid doses and corresponding prednisolone conversion factors.

	Equivalent glucocorticoid dose	Prednisolone conversion factor
Hydrocortisone	20	0.25
Methylprednisolone	4	1.25
Prednisolone	5	1
Prednisone	5	1
Dexamethasone	0.75	6.67
Betamethasone	0.6	8.33
Triamcinolone	4	1.25

Cumulative dose calculation:

The cumulative prednisolone-equivalent dose was calculated by multiplication of the number of pills/injections, dose per pill/injection, and prednisolone conversion factor for the drug of interest.

© 2025 Laugesen K et al. *JAMA Network Open.*

eTable 4. Parental and pregnancy characteristics of exposed and unexposed siblings. Characteristics of the unexposed cohorts are shown before and after weighting[1].

	N (%)					
	Risk of preterm delivery			Autoimmune or inflammatory disorders		
	Exposed siblings	Unexposed siblings (unweighted)	Unexposed siblings (weighted)	Exposed siblings	Unexposed siblings (unweighted)	Unexposed siblings (weighted)
All births	139	129	118	2437 (100)	2805 (100)	2289 (100)
Offspring sex, male	68 (49)	77 (60)	59 (50)	1258 (52)	1469 (52)	1209 (52)
Year of conception						
1999–2002	25 (18)	24 (19)	20 (17)	360 (15)	339 (12)	350 (15)
2003–2006	54 (39)	44 (34)	49 (41)	589 (24)	630 (22)	561 (25)
2007–2010	21 (15)	23 (18)	16 (14)	765 (31)	848 (30)	689 (30)
2011–2015	39 (27)	38 (29)	33 (28)	723 (30)	988 (35)	689 (30)
**Maternal characteristics**						
Age at birth (y), median and IQR	31 (26–35)	30 (27–33)	31 (26–35)	33 (29–37)	33 (30–37)	33 (29–37)
Parity						
0	52 (37)	54 (42)	41 (36)	1258 (52)	2113 (87)	1199 (52)
≥1	87 (63)	72 (56)	77 (65)	1147 (47)	1925 (69)	1090 (48)
Missing	0 (0)	NA	0 (0)	32 (1.3)	30 (1.1)	0 (0)
Country of origin						
Danish	103 (74)	91 (71)	87 (73)	2113 (87)	2413 (86)	2016 (88)
Missing	0 (0)		0 (0)	13 (0.3)	16 (0.6)	0 (0)
Highest educational level						
Low	31 (22)	30 (23)	27 (22)	409 (17)	520 (19)	407 (18)
Moderate	38 (27)	38 (29)	35 (30)	972 (40)	1067 (38)	905 (40)
High	61 (44)	52 (40)	54 (46)	994 (41)	1130 (40)	977 (43)
Missing	9 (6.4)	8 (6.2)	0 (0)	62 (2.5)	88 (3.1)	0 (0)
Marital status						
Married/civil partnership	69 (50)	66 (51)	62 (52)	1440 (59)	1798 (64)	1358 (59)
Missing	0 (0)	0 (0)	0 (0)	14 (0.6)	17 (0.6)	0 (0)
BMI (kg/m^2), median IQR						
<18.5	13 (10)	8 (7.6)	13 (9.5)	88 (4.2)	96 (3.9)	84 (4.3)
18.5–24	74 (58)	59 (56)	58 (59)	1199 (58)	1363 (55)	1105 (57)
25–29	17 (13)	19 (18)	13 (14)	410 (20)	546 (22)	400 (21)
≥30	15 (12)	13 (12)	11 (11)	288 (14)	353 (14)	262 (14)
Missing (2004 onward)	8 (6.3)	6 (5.7)	NA	92 (4.4)	108 (4.4)	88 (4.5)

© 2025 Laugesen K et al. *JAMA Network Open.*

Not recorded before 2004	12 (100)	24 (100)	20 (100)	360 (100)	339 (100)	350 (100)
Smoking during pregnancy						
Yes	17 (12)	16 (12)	13 (11)	366 (15)	400 (14)	358 (16)
Obstructive pulmonary disease	29 (21)	27 (21)	26 (22)	1298 (53)	1641 (59)	1236 (54)
Inflammatory bowel disease	NA	NA	NA	197 (8.0)	196 (7.0)	192 (8.4)
Rheumatic disease	NA	NA	NA	252 (10)	235 (8.4)	230 (10)
Renal disease	NA	NA	NA	109 (4.5)	134 (4.8)	107 (4.7)
Skin disease	NA	NA	NA	29 (1.2)	35 (1.3)	30 (1.3)
Neurodevelopmental disorders	NA	NA	NA	19 (0.8)	23 (0.8)	16 (0.7)
Mood, anxiety, or stress-related disorders	32 (23)	28 (22)	25 (22)	476 (20)	598 (21)	465 (20)
Schizophrenia spectrum disorders	6 (4.3)	NA	NA	89 (3.7)	106 (3.8)	93 (4.0)
Substance use disorders	NA	NA	NA	21 (0.9)	22 (0.8)	18 (0.8)
PCOS	NA	NA	NA	56 (2.3)	69 (2.5)	52 (2.3)
Co-medication use during pregnancy						
Other immunosuppressive agents	NA	NA	NA	95 (3.9)	26 (0.9)	87 (3.8)
NSAIDs	NA	6 (4.6)	NA	86 (3.5)	39 (1.4)	72 (3.2)
Antiepileptic medications	NA	NA	NA	11 (0.4)	7 (0.3)	9 (0.4)
Opioids	NA	NA	NA	38 (1.6)	30 (1.1)	39 (1.7)
Antidepressants	NA	NA	NA	57 (2.3)	34 (1.2)	61 (2.7)
Antipsychotics	NA	NA	NA	7 (0.3)	5 (0.2)	NA
Stimulants	NA	NA	NA	NA	NA	NA
**Paternal characteristics**						
Age at birth (y), median and IQR	33 (29–37)	32 (29–36)	33 (29–37)	33 (29–37)	33 (30–37)	33 (29–37)
Neurodevelopmental disorders	NA	NA	NA	25 (1.0)	23 (0.8)	26 (1.1)
Mood, anxiety, or stress-related disorders	11 (7.9)	13 (10)	10 (8.5)	246 (10)	309 (11)	239 (10)
Schizophrenia spectrum disorders/psychoses	NA	NA	NA	69 (2.8)	78 (2.8)	65 (2.9)
Substance use disorders	NA	NA	NA	30 (1.2)	36 (1.3)	34 (1.5)
**Pregnancy complications**						
Multiple pregnancy	28 (20)	11 (8.5)	24 (21)	373 (15)[2]	249 (9.0)	226 (9.9)[2]
Gestational diabetes	NA	NA	NA	82 (3.4)[2]	82 (3.4)	64 (2.8)[2]
Preeclampsia	10 (7.2)	NA	6 (4.8)	137 (5.6)[2]	102 (3.6)	102 (4.5)[2]
Infections	32 (23)	26 (20)	24 (21)	468 (19)[2]	738 (26)	454 (20)[2]

[1]Standardized differences are all ≤0.1. They are not shown because of Danish legislation regarding individual-level data.

2Variables considered potential mediators (Supplemental Figure 2), thus not included in the PS-weighting. The variables are not expected to be balanced after weighting.

© 2025 Laugesen K et al. *JAMA Network Open.*

Abbreviations: BMI, body mass index; IQR, interquartile range; NSAIDs, nonsteroidal anti-inflammatory drugs; SGA, small for gestational age. Highest educational level at birth: low (primary and lower secondary education), medium (upper secondary education or professional degree), and high (university education at bachelor's degree level or higher). NA: not shown due to Danish legislation on presenting micro data.

© 2025 Laugesen K et al. *JAMA Network Open.*

eTable 5. Numbers at risk and numbers of outcomes according to exposure in unweighted and weighted cohorts. The numbers support Figure 2 and 3.

	Numbers				
	**At risk**	**Autism spectrum disorders**	**Intellectual disabilities**	**ADHD**	**Mood, anxiety, or stress-related disorders**
**Risk of preterm delivery**					
Exposed	3659	104	35	92	82
Unexposed (unweighted)	27,859	767	254	902	916
Unexposed (weighted)	3349	76	22	66	69
**Autoimmune or inflammatory disorders**					
Exposed	6453	154	36	164	167
Unexposed (unweighted)	282,294	5999	1578	7412	7640
Unexposed (weighted)	6010	113	26	118	129
Former use (unweighted)	45,576	960	218	1136	1248
Former use (weighted)	6022	114	24	130	135
Cumulative dose (mg peq)					
<250	2344	60	16	81	73
Unexposed (weighted)	2199	46	12	53	58
250-499	1025	21	NA	16	19
Unexposed (weighted)	974	16	3931	18	18
=>500	3084	73	17	67	75
Unexposed (weighted)	2867	50	10	48	54

Abbreviations: ADHD, attention deficit hyperactivity disorder; peq, prednisolone equivalents.

© 2025 Laugesen K et al. *JAMA Network Open.*

eTable 6. Results of sensitivity analyses.

	Adjusted 15-year relative risks (95% confidence intervals)			
**Outcomes**	Sibling analysis	Outcome detected by hospital code or prescription medication	Restricted to singleton births	Active comparator
**Risk of preterm delivery**				
Autism	NA	Not relevant	1.5 (1.0 to 1.9)	Not relevant
Intellectual disability	NA	Not relevant	1.2 (0.7 to 1.8)	Not relevant
ADHD	NA	1.5 (1.2 to 1.9)	1.3 (0.9 to 1.7)	Not relevant
Mood, anxiety, or stress-related disorders	NA	1.4 (1.1 to 1.9)	1.5 (1.1 to 2.1)	Not relevant
Composite outcome	1.4 (0.5 to 3.9)	Not relevant	Not relevant	Not relevant
**Autoimmune or inflammatory disorders**				
Autism	NA	Not relevant	1.2 (1.0 to 1.5)	NA
Intellectual disability	NA	Not relevant	1.4 (0.8 to 2.0)	NA
ADHD	NA	1.3 (1.1 to 1.5)	1.3 (1.1 to 1.6)	NA
Mood, anxiety, or stress-related disorders	NA	1.4 (1.2 to 1.7)	1.4 (1.1 to 1.7)	NA
Composite outcome	1.3 (1.0 to 1.6)	Not relevant	Not relevant	1.7 (1.1 to 3.5)

Notes: Adjusted for year of conception; parity; maternal and paternal age at conception; maternal smoking during pregnancy; specific maternal autoimmune or inflammatory disorders; maternal and paternal neurodevelopmental disorders; maternal and paternal mood, anxiety, and stress-related disorders; maternal and paternal schizophrenia spectrum disorders; maternal and paternal substance use disorders; maternal polycystic ovarian syndrome; maternal use of co-medications during pregnancy, including nonsteroidal anti-inflammatory drugs, other immunosuppressive agents, opioids, antiepileptic medications, antidepressants, antipsychotics, and stimulants; highest maternal educational level at conception; maternal country of origin; and civil status. For offspring born to women at risk of preterm delivery, we additionally adjusted for singleton/multiple pregnancy, gestational diabetes, preeclampsia, and maternal infections during pregnancy.
Abbreviation: ADHD, attention deficit hyperactivity disorder; NA, not applicable due to low number of outcomes.

© 2025 Laugesen K et al. *JAMA Network Open.*

eTable 7. Results of sensitivity analyses.

	Adjusted 15-year relative risks (95% confidence intervals)			
**Outcomes**	Restricted to offspring born SGA	SGA included as covariate	Restriction to risk of preterm birth only	Restriction to autoimmune or inflammatory disorders only
**Risk of preterm delivery**				
Autism	1.5 (0.9 to 2.6)	1.4 (1.1 to q.7)	1.4 (1.11 to 1.8)	Not relevant
Intellectual disability	1.3 (0.6 to 2.9)	1.5 (1.1 to 2.5)	1.7 (1.1 to 2.7)	Not relevant
ADHD	1.3 (0.6 to 2.1)	1.3 (1.1. to 1.6)	1.4 (1.1 to 1.7)	Not relevant
Mood, anxiety, or stress-related disorders	1.4 (0.6 to 2.1)	1.3 (0.9 to 1.4)	1.3 (0.9 to 1.6)	Not relevant
**Autoimmune or inflammatory disorders**				
Autism	Not relevant	Not relevant	Not relevant	1.3 (1.0 to 1.6)
Intellectual disability	Not relevant	Not relevant	Not relevant	1.4 (0.9 to 2.4)
ADHD	Not relevant	Not relevant	Not relevant	1.3 (1.0 to 1.5)
Mood, anxiety, or stress-related disorders	Not relevant	Not relevant	Not relevant	1.4 (1.1 to 1.8)

Notes: Adjusted for year of conception; parity; maternal and paternal age at conception; maternal smoking during pregnancy; specific maternal autoimmune or inflammatory disorders; maternal and paternal neurodevelopmental disorders; maternal and paternal mood, anxiety, and stress-related disorders; maternal and paternal schizophrenia spectrum disorders; maternal and paternal substance use disorders; maternal polycystic ovarian syndrome; maternal use of co-medications during pregnancy, including nonsteroidal anti-inflammatory drugs, other immunosuppressive agents, opioids, antiepileptic medications, antidepressants, antipsychotics, and stimulants; highest maternal educational level at conception; maternal country of origin; and civil status. For offspring born to women at risk of preterm delivery, we additionally adjusted for singleton/multiple pregnancy, gestational diabetes, preeclampsia, and maternal infections during pregnancy.
Abbreviation: ADHD, attention deficit hyperactivity disorder; NA, not applicable due to low number of outcomes; SGA, small for gestational age.

© 2025 Laugesen K et al. *JAMA Network Open.*

eTable 8. Results of sensitivity analyses.

	Adjusted 15-year relative risks (95% confidence intervals)		
**Outcomes**	Exposed to prednisolone only	Exposed to prednisone only	Exposed to antenatal betamethasone only
**Risk of preterm delivery**			
Autism	Not relevant	Not relevant	1.5 (1.2 to 1.9)
Intellectual disability	Not relevant	Not relevant	1.3 (0.8 to 1.8)
ADHD	Not relevant	Not relevant	1.3 (1.0 to 1.7)
Mood, anxiety, or stress-related disorders	Not relevant	Not relevant	1.5 (1.1 to 2.0)
**Autoimmune or inflammatory disorders**			
Autism	1.4 (1.2 to 1.8)	1.9 (1.2 to 3.1)	Not relevant
Intellectual disability	1.4 (0.9 to 2.0)	1.8 (0.6 to 5.0)	Not relevant
ADHD	1.3 (1.1 to 1.5)	1.9 (1.2 to 3.1)	Not relevant
Mood, anxiety, or stress-related disorders	1.4 (1.2 to 1.8)	1.6 (0.9 to 2.7)	Not relevant

Notes: Adjusted for year of conception; parity; maternal and paternal age at conception; maternal smoking during pregnancy; specific maternal autoimmune or inflammatory disorders; maternal and paternal neurodevelopmental disorders; maternal and paternal mood, anxiety, and stress-related disorders; maternal and paternal schizophrenia spectrum disorders; maternal and paternal substance use disorders; maternal polycystic ovarian syndrome; maternal use of co-medications during pregnancy, including nonsteroidal anti-inflammatory drugs, other immunosuppressive agents, opioids, antiepileptic medications, antidepressants, antipsychotics, and stimulants; highest maternal educational level at conception; maternal country of origin; and civil status. For offspring born to women at risk of preterm delivery, we additionally adjusted for singleton/multiple pregnancy, gestational diabetes, preeclampsia, and maternal infections during pregnancy.
Abbreviation: ADHD, attention deficit hyperactivity disorder; NA, not applicable due to low number of outcomes.

© 2025 Laugesen K et al. *JAMA Network Open.*

eTable 9. Results of subgroup analyses.

	Adjusted 15-year relative risks (95% confidence intervals)					
**Outcomes**	Males	Females	Obstructive pulmonary disease	Inflammatory bowel disease	Rheumatic disease	Renal disease
**Risk of preterm delivery**						
Autism	1.5 (1.2 to 2.1)	1.5 (0.9 to 2.4)	Not relevant	Not relevant	Not relevant	Not relevant
Intellectual disability	1.1 (0.7 to 1.8)	1.8 (0.9 to 3.8)	Not relevant	Not relevant	Not relevant	Not relevant
ADHD	1.4 (1.0 to 1.9)	1.4 (0.8 to 2.1)	Not relevant	Not relevant	Not relevant	Not relevant
Mood, anxiety, or stress-related disorders	1.2 (0.8 to 1.8)	1.9 (1.3 to 2.7)	Not relevant	Not relevant	Not relevant	Not relevant
**Autoimmune or inflammatory disorders**						
Autism	1.2 (1.0 to 1.4)	1.5 (0.9 to 2.2)	1.5 (1.2 to 1.9)	1.4 (0.5 to 2.8)	NA	NA
Intellectual disability	1.5 (0.9 to 2.4)	1.2 (0.7 to 2.2)	1.7 (1.0 to 2.5)	1.7 (0.4 to 2.7)	NA	NA
ADHD	1.2 (1.0 to 1.4)	1.2 (1.0 to 1.4)	1.4 (1.1 to 1.7)	1.2 (0.6 to 2.2)	NA	NA
Mood, anxiety, or stress-related disorders	1.3 (1.0 to 1.6)	1.3 (1.0 to 1.6)	1.6 (1.0 to 2.4)	1.6 (0.7 to 2.8)	NA	NA
Composite outcome	1.4 (1.2-1.5)	1.4 (1.2-1.5)	1.4 (1.2-1.6)	1.3 (0.8-1.7)	1.3 (0.8-1.6)	1.8 (1.2-2.8)

Notes: Adjusted for year of conception; parity; maternal and paternal age at conception; maternal smoking during pregnancy; specific maternal autoimmune or inflammatory disorders; maternal and paternal neurodevelopmental disorders; maternal and paternal mood, anxiety, and stress-related disorders; maternal and paternal schizophrenia spectrum disorders; maternal and paternal substance use disorders; maternal polycystic ovarian syndrome; maternal use of co-medications during pregnancy, including nonsteroidal anti-inflammatory drugs, other immunosuppressive agents, opioids, antiepileptic medications, antidepressants, antipsychotics, and stimulants; highest maternal educational level at conception; maternal country of origin; and civil status. For offspring born to women at risk of preterm delivery, we additionally adjusted for singleton/multiple pregnancy, gestational diabetes, preeclampsia, and maternal infections during pregnancy.
Abbreviation: ADHD, attention deficit hyperactivity disorder; NA, not applicable due to low numbers of outcomes.

© 2025 Laugesen K et al. *JAMA Network Open.*

eTable 10. Adjusted 15-year relative risks and 95% confidence intervals for exposed offspring *vs.* all unexposed offspring from the general population.

	Autism spectrum disorders	Intellectual disability	ADHD	Mood, anxiety, or stress-related disorders
**Risk of preterm delivery**				
Exposed vs. all unexposed in the general population	1.9 (1.6 to 2.5)	2.0 (1.4 to 2.7)	1.6 (1.2 to 1.9)	1.9 (1.5 to 2.9)
**Autoimmune or inflammatory disorders**				
Exposed vs. all unexposed in the general population	1.3 (1.1 to 1.7)	1.4 (1.0 to 1.9)	1.4 (1.2 to 1.6)	1.6 (1.3 to 2.0)

Notes: Adjusted for year of conception; parity; maternal and paternal age at conception; maternal smoking during pregnancy; maternal and paternal neurodevelopmental disorders; maternal and paternal mood, anxiety, and stress-related disorders; maternal and paternal schizophrenia spectrum disorders; maternal and paternal substance use disorders; maternal polycystic ovarian syndrome; maternal use of co-medications during pregnancy, including nonsteroidal anti-inflammatory drugs, other immunosuppressive agents, opioids, antiepileptic medications, antidepressants, antipsychotics, and stimulants; highest maternal educational level at conception; maternal country of origin; and civil status. For offspring born to women at risk of preterm delivery, we additionally adjusted for singleton/multiple pregnancy, gestational diabetes, preeclampsia, and maternal infections during pregnancy. Abbreviation: ADHD, attention deficit hyperactivity disorder.

© 2025 Laugesen K et al. *JAMA Network Open.*
