# Supplementary material for: Mental Disorders Among Offspring Prenatally Exposed to Systemic Glucocorticoids
Source: JAMA Netw Open. 2025 Jan 3;8(1):e2453245. doi: 10.1001/jamanetworkopen.2024.53245 (PMC11699534; doi:10.1001/jamanetworkopen.2024.53245)
Supplement: Supplement 2. — Data Sharing Statement [file jamanetwopen-e2453245-s002.pdf]

Data Sharing Statement

Data

Data available: No

Additional Information

Explanation for why data not available: Data presented in this study were obtained from Danish registries. Because of Danish data protection rules, we are not allowed to share individual-level data. Other researchers meeting the requirements set by the data providers could obtain similar data.
